# Gene-Exercise Interactions in Amyloid Metabolism and Clearance: Implications for Alzheimer’s Disease

**DOI:** 10.3390/ijms26199816

**Published:** 2025-10-09

**Authors:** Maria Francesca Astorino, Giovanni Luca Cipriano, Ivan Anchesi, Maria Lui, Ivana Raffaele, Marco Calabrò, Concetta Crisafulli

**Affiliations:** 1 Department of Biomedical and Dental Sciences and Morpho-Functional Imaging—BIOMORF, University of Messina, 98125 Messina, Italy; mastorino@unime.it (M.F.A.); ccrisafulli@unime.it (C.C.); 2 IRCCS Centro Neurolesi “Bonino-Pulejo”, Via Provinciale Palermo, Contrada Casazza, 98124 Messina, Italy; giovanniluca.cipriano@irccsme.it (G.L.C.); ivan.anchesi@irccsme.it (I.A.); maria.lui@irccsme.it (M.L.); ivana.raffaele@irccsme.it (I.R.)

**Keywords:** Alzheimer’s Disease, genetics, physical exercise, amyloid metabolism, amyloid clearance

## Abstract

Alzheimer’s disease (AD), the most prevalent form of dementia, poses a critical global health challenge as its incidence rises with aging populations. Despite extensive research into its genetic and molecular underpinnings, effective therapeutic strategies remain limited. Growing evidence suggests that physical exercise may offer neuroprotective benefits, potentially mitigating AD progression through multifactorial mechanisms. This review synthesizes current findings on the interplay between aerobic exercise and AD pathophysiology, with a focus on amyloid-β (Aβ) metabolism, gene expression, and neuroinflammation. We explore how exercise influences Aβ clearance, modulates amyloid precursor protein (APP) processing, and impacts the activity of key enzymes such as secretases and neprilysin. Further, we highlight the gene–exercise crosstalk identified through transcriptomic data, particularly in the entorhinal cortex—an early site of Aβ deposition. Our analysis also discusses how exercise-induced modulation of molecular pathways—including mitochondrial function, oxidative stress responses, and neuroinflammatory cascades—may confer cognitive resilience. By integrating molecular, genetic, and systems biology data, this review underscores the potential of structured physical activity as a non-pharmacological intervention to delay or attenuate AD pathology. These insights support a precision medicine approach, which combines lifestyle interventions with molecular profiling, to improve prevention strategies and therapeutic outcomes in AD.

## 1. Introduction

**Figure 1:**
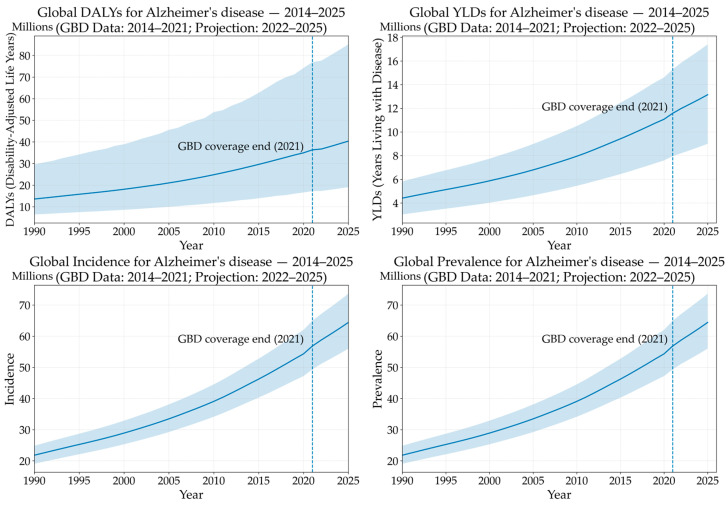
AD trends from 1990 to 2025.

As lifestyles evolve and life expectancy rises, the prevalence of neurodegenerative disorders—such as Alzheimer’s disease (AD), Parkinson’s disease (PD), and prion diseases—has significantly increased worldwide. The World Health Organization (WHO) describes dementia as a broad term encompassing multiple pathological conditions that gradually impair memory, cognitive abilities, and behavior, ultimately disrupting an individual’s capacity to perform daily tasks. In 2019, it was estimated that 57.4 million people worldwide are affected by dementia [1]. Further projections indicate that this number will rise to 83.2 million by 2030, 116.0 million by 2040, and 152.8 million by 2050 [1]. Among these, the data on AD is particularly concerning, earning it the definition of “plague of the 21st century” (Figure 1). AD is one of the fastest rising diseases among the leading 50 causes of decreased life expectancy. This disease is estimated to account for almost 60–80% of all dementia cases [2], with a prevalence of over ~52 million individuals, and an incidence rate of 1% at age 60 which doubles every five years [3].

Graphs reported show the trends of AD epidemiological data, including disability-adjusted life years, years lived with disability, prevalence, and incidence. Graphs were obtained using the GBD results tool—GBD2021 (https://vizhub.healthdata.org/gbd-results/, accessed on 24 September 2025) up to 2021, which was the year of the latest GBD report [4]. Data from 2022 to 2025 were added as projection on the basis of the previous data from GBD. The dashed line marks the transition to projected data. The latest data from GBD are also reported in Table 1.

Research in this area has detailed several aspects about the biological and environmental underpinnings of AD. However, the precise mechanisms behind the disease and its treatment remain largely unknown [5]. Understanding the molecular origins and mechanisms of AD is essential for developing targeted therapies and potential cures. It has been observed that genetic factors likely play a pivotal role in AD, with specific genes that have been linked to both early-onset and late-onset forms of the disease. Early-onset Alzheimer’s disease (EOAD) is primarily caused by autosomal dominant mutations in three key genes: amyloid precursor protein (*APP*), presenilin 1 (*PSEN1*), and presenilin 2 (*PSEN2*). These mutations promote abnormal amyloid-beta (Aβ) accumulation, which is a hallmark of AD pathology [6]. In contrast, late-onset Alzheimer’s disease (LOAD) is associated with a more complex inheritance pattern, where the apolipoprotein E (*APOE*) gene plays a significant role. The *APOE* ε4 allele substantially increases the risk of LOAD by impairing Aβ clearance and exacerbating neuroinflammation [7].

Genome-wide association studies (GWAS) have broadened our understanding of AD genetics, identifying additional risk loci, including *ABCA7*, *BIN1*, *CLU*, *CR1*, and *TREM2* [8]. Variants in these genes disrupt critical biological pathways such as lipid metabolism, immune response, and synaptic function, all of which contribute to AD pathogenesis [7,8].

A crucial pathological feature of AD involves the conversion of normal soluble proteins into amyloid fibrils, a process now recognized as an intrinsic property of polypeptide chains [9]. Mutations in amyloidogenic proteins, such as Aβ, α-synuclein (SNCA), and prion proteins, increase the risk of neurodegenerative diseases, including AD. Protein misfolding is influenced by multiple factors, including environmental changes (e.g., pH, temperature, and protein concentration), post-translational modifications, increased degradation rates, trafficking errors, loss of binding partners, and oxidative damage. These factors may act independently or synergistically, accelerating disease progression [3].

Importantly, increasing evidence supports the role of environmental factors, in addition to genetic factors in AD. Notably, particular focus has been posed to the role of physical exercise in preventing and slowing the progression of AD. Regular physical activity enhances cerebral blood flow, promotes neurogenesis, and reduces inflammation, contributing to improved cognitive function and delayed disease onset [10]. Exercise also mitigates oxidative stress and enhances the synthesis of neurotrophic factors, which are essential for neuronal survival and plasticity [11]. Furthermore, physical activity has been shown to modulate Aβ metabolism and reduce Aβ plaques accumulation, a hallmark of AD [12]. Emerging studies highlight a direct link between physical exercise and the reduction in Aβ pathology. According to the literature, exercise influences Aβ clearance mechanisms and decreases Aβ plaques deposition [13,14,15]. This effect is believed to be mediated by enhanced enzymatic degradation of Aβ and improved blood–brain barrier (BBB) function, facilitating Aβ removal from the brain [16]. Additionally, exercise-induced modulation of mitochondrial function through pathways such as SIRT1-FOXO1/3-PINK1-PRKN enhances cellular energy metabolism and promotes Aβ clearance [17].

Interestingly, research data also highlight the impact of exercise on brain plasticity and cognitive resilience. For instance, aerobic and resistance training programs can improve memory and executive functions in individuals at risk for AD [18]. Additionally, the modulation of the gut–brain axis through physical exercise appears to influence neuroinflammatory pathways and cognitive health, offering a novel avenue for AD prevention [19].

In light of this evidence, this review discusses the impact of aerobic exercise on AD, examining its effects on cognition, neurobiological mechanisms, and potential clinical applications. Additionally, we will explore if and how physical exercise may mitigate Aβ accumulation while modulating the associated biological cascades. The rationale behind this focus is that, despite significant advances in AD genetics research, a substantial portion of the genetic architecture underlying the disease remains unexplained. The critical role of Aβ accumulation has led us to hypothesize that, regardless of the specific biological pathway involved in AD, these mechanisms ultimately converge on Aβ deposition. Consequently, investigating the biological functions related to Aβ may have the potential to increase our understanding of AD as a whole and provide potential new molecular targets for treatment.

Many mechanistic studies investigating the interactions between physical activity and amyloid metabolism have used aerobic paradigms, for example, treadmill running, voluntary wheel running, or structured endurance training. Consequently, most of the detailed molecular evidence, such as effects on glymphatic clearance, SIRT1-mediated mitophagy, mitochondrial proteostasis, and enzyme-mediated Aβ degradation, derives from aerobic interventions [12,18,20,21]. By consolidating findings from the recent literature and linking them with biological data, this review will contribute to a better understanding of how structured physical activity, in relation to genetic factors, can be leveraged to provide new information to develop approaches for AD symptoms management and for improving patients’ quality of life.

## 2. Materials and Methods

This narrative review followed the PRISMA checklist for the literature research; the complete study selection process is detailed in the PRISMA flowchart (Supplementary Figure S1). We searched PubMed and Google Scholar for publications from their inception until 31 January 2025. The keywords used were any physical exercise-related words (“Exercise” OR “Physical exercise” OR “Aerobic”), Alzheimer’s disease (“Alzheimer” OR “Alzheimer’s Disease”), amyloid-β (“Amyloid-β” or “Aβ”), molecular pathways (“Clearance” OR “Regulation”), genes (“Alzheimer’s Genes” OR “Aβ Genes”), and human (“human” OR “patients”).

Our inclusion criteria focused on studies, including animal models and in silico data, that examined the effects of physical exercise on Aβ regulation or the expression of AD-related genes.

To construct similarity networks, we mined protein–protein interaction data from the STRING and Gene Ontology databases. For differential expression analysis, we retrieved data from the EMBL-EBI Expression Atlas [22], focusing on the entorhinal cortex of AD patients versus controls. Expression variation was calculated as a ratio of the raw expression in AD to that in controls for each gene.

## 3. Environmental Factors in AD 

Several risk factors for AD have been identified, including nonmodifiable ones such as aging, as well as modifiable factors like environmental pollution and diet, which can be targeted for preventive strategies [1]. Researchers categorized 14 factors that may increase the risk of dementia based on different life stages [23]. These include early-life factors (limited education), midlife factors (hearing loss, high LDL cholesterol, depression, traumatic brain injury, physical inactivity, diabetes, smoking, hypertension, obesity, and excessive alcohol consumption), and late-life factors (social isolation, air pollution, and vision impairment) [23]. Overall, addressing these 14 risk factors could potentially prevent or delay up to 45% of dementia cases.

Recognizing that most diseases—aside from single-gene disorders—result from complex interactions between genetic, environmental, and other risk factors is crucial [24]. To improve health outcomes, various strategies should be explored, such as maintaining a nutritious diet and utilizing nutraceuticals aimed at key elements like oxidative stress, inflammation, and mitochondrial function [1].

It is equally important to evaluate the potential effects of environmental contaminants—such as air pollution and prolonged pesticide exposure—since these factors can significantly influence health [25]. Exposure to neurotoxic metals, which can accumulate in the brain, exacerbate oxidative stress, and contribute to neuronal damage [26]. The involvement of copper, or zinc in Aβ plaque formation, along with oxidative stress driven by metal catalysis, is implicated in the pathogenesis of AD and may result from impaired metal homeostasis [27]. Also, diet plays a significant role in AD risk. High-fat and high-caloric diets are associated with an increased likelihood of AD, while the consumption of fish and whole grains may have protective effects [26]. Finally, physical activity, as it will be discussed in the next section, is also crucial in delaying cognitive decline and reducing AD risk.

### How Lifestyle Factors Influence Alzheimer Disease

A healthy lifestyle, considered as a composite score, is significantly associated with a reduced risk of AD. Moreover, adhering to a healthy lifestyle is linked to a slower rate of memory decline [28,29]. Longitudinal data suggest that physical activity and a balanced diet are associated with a lower incidence of AD [28].

In particular, physical exercise has emerged as a promising non-pharmacological intervention for AD prevention and management. Regular physical activity is associated with improved cognitive function, increased hippocampal volume, and enhanced cerebral blood flow [18].

Physical inactivity leads to approximately 5 million deaths worldwide each year due to noncommunicable diseases [30]. Aerobic exercise has been studied to mitigate the effects of aging on cognitive function [31]. From the first evidence, dating back to the 1970s, it showed that middle-aged athletes outperformed their sedentary peers in cognitive tasks requiring psychomotor skills. More recent research found that physically active middle-aged individuals performed significantly better on memory tests than their sedentary counterparts [32]. A study [33] found that a year of moderate-intensity exercise increased hippocampal volume and improved spatial memory in older adults.

Exercise modulates Aβ metabolism, reduces inflammation, and promotes neurogenesis [32]. In a comparative study using animal models, sustained physical exercise reduced Aβ plaque burden and preserved cognitive function, emphasizing the therapeutic potential of long-term physical activity in AD [34]. Therefore, lifestyle factors, Aβ accumulation, and physical exercise are interconnected elements in the pathogenesis and potential mitigation of AD and amyloid pathology remains a central focus for therapeutic development.

## 4. Amyloid Dis-Equilibrium in Alzheimer’s Disease and Physical Exercise Influences

Aβ plays a crucial role in AD [35]. Its misfolding and aggregation are typical in this disease and usually triggers toxic effects on neurons [36]. The process begins with the sequential cleavage of APP by β- and γ-secretases, leading to the formation of Aβ peptides. These peptides tend to aggregate, through a complex, multistep process, into oligomers and then insoluble fibrils, which form extracellular plaques in the human brain, characteristic of AD pathology [37]. Interestingly, the current literature suggests that Aβ oligomers, rather than mature fibrils, represent the primary drivers of neurotoxicity, contributing to cognitive decline and neuronal death [3].

In detail, Aβ peptides assemble into small, soluble oligomeric structures that serve as nuclei for rapid fibril growth [38,39]. These oligomers interact with metal ions like zinc and copper, exacerbating oxidative damage and leading to neuronal death [40]. The rate-limiting step in fibril formation involves the assembly of monomeric peptides into oligomers, a process influenced by N-terminal modifications. In physiological conditions, Aβ structures are known to contribute to melanosome biogenesis and long-term memory formation [41,42]. However, in pathological conditions, Aβ-Aβ oligomers/fibrils equilibrium drastically skews toward aggregation. Additionally, the Aβ peptides can also bind to other proteins, including tau and α-synuclein, leading to cross-seeding, where one misfolded protein accelerates the aggregation of another [43]. In this context, physical exercise has emerged as a potential modulator of Aβ equilibrium through various mechanisms affecting Aβ production, accumulation, and clearance [16], ultimately ameliorating cognitive decline. In the following sections we will discuss the known effects of exercise on these processes.

### 4.1. Effects of Physical Exercise on Amyloid Production

The literature data highlighted the beneficial effects of physical exercise in countering Aβ overproduction: animal studies suggest that treadmill exercise may decrease Aβ deposits in the hippocampus by modulating APP metabolism [44]. Moreover, systematic reviews and meta-analyses in animal models discussed how chronic physical exercise, including treadmill running and swimming, reduces Aβ levels, particularly Aβ1-42, through the modulation of amyloidogenic pathways [45]. In humans, resistance training has also been linked to decreased brain Aβ accumulation, highlighting its potential neuroprotective effects [46]. Nevertheless, despite having a potent beneficial effect on cognitive function in older adults with elevated Aβ levels, some studies showed no significant effects on Aβ accumulation [47]. The lack of consistent findings may be attributable to the influence of factors such as exercise intensity, duration, and participant characteristics on the impact of physical activity in reducing Aβ accumulation.

Focusing on the biological and genetic aspects of physical exercise influence on Aβ production, particular emphasis should be placed on α-secretase, β-secretase, and γ-secretase, well-known proteases strictly involved in Aβ generation [48]. Investigating the regulatory network associated with these elements, may help characterize how physical exercise affects Aβ production process [49,50,51]. Due to their pleiotropic role in numerous biological processes, the modulation of these enzymes’ expression is likely modulated by numerous elements. To constrain the number of elements to investigate, in this review we mainly focused on the regulatory genes that were also dysregulated in AD patients, based on the data contained in the Expression Atlas from EMBL-EBI database. Specifically, according to our selection (see methods section, we selected genes that were significantly dysregulated in the entorhinal cortices of AD patients compared to controls. Table 2 reports the genes down- and up-regulated associated with secretases network.

The selected genes were further investigated to characterize their role in AD. Potential associations with physical exercise were also highlighted. Table 3 reports a summary of the data.

Among the genes reported, we focused on the ones showing a correlation to both AD and physical activity. The other genes reported in Table 3, to the best of our knowledge, were never investigated in association with physical activity. Future studies may shed light on their possible association with exercise. Among them, the *APBB2* gene (Amyloid Beta Precursor Protein Binding Family B Member 2), encoding for FE65L1 protein, can interact with the intracellular domain of APP and is critical for synaptic development [54]. It has been observed that FE65L1 can enhance γ-secretase processing of APP, promoting the production of the amyloidogenic C-Terminal fragment (among the others) [73]. Thus, its increased expression may skew the balance of APP processing towards the amyloidogenic pathway. Interestingly, studies on the effect of physical exercise on this gene’s expression highlighted how *APPB2* is down-regulated in subjects engaged in high and moderate physical activity [55]. This data provides a potential link between the beneficial effects of physical exercise and the amelioration of AD from a molecular point of view, as exercise-induced decrease in *APPB2* expression, removes a factor involved in Aβ production.

*ARF1*, also known as ARF GTPase 1 and ADP-Ribosylation Factor 1, encodes for a protein critical for APP maturation in neuronal cells, mainly through its action on β-secretase. Although it does not directly affect this secretase, *ARF1* encoded protein appears to be essential for the correct transport of BACE1 to the cell membrane [56]: depletion of this enzyme has been associated with increased BACE1 concentration at the TGN and decreased concentration at the cell surface. Interestingly, the literature data highlighted how this change in BACE1 distribution, positively influences Aβ production [56], suggesting an amyloidogenic role for *ARF1* down-regulation.

Notably, it has been observed that *ARF1* can be up-regulated by endurance exercise in human muscles. Also, a single acute exercise significantly increased both *ARF1* mRNA and encoded protein levels in human muscle biopsies [57]. Although this effect in central nervous system (CNS) is yet to be investigated, this evidence provides another possible molecular bridge to explain the beneficial effects of physical exercise on Aβ metabolism.

*MAP2* encoded protein showed some correlation with γ-secretase activity and the gene is down-regulated in AD. However, a deeper focus on the literature data highlights how this gene’s expression is a consequence of AD evolution rather than a modulator. Indeed, several studies highlighted how Aβ accumulation in neurons coincided with a progressive decline of *MAP2* encoded protein, which in turn disrupts neuronal morphology and is likely linked to neuronal loss [61]. Interestingly, some of the literature data correlate physical activity on an up-regulation of *MAP2* expression [62]. Thus, while not targeting a primary effector of AD, physical activity may counter neuronal loss through the up-regulation of this gene.

Shifting focus to inflammatory pathways, especially on NF-κB signaling, the literature evidence highlighted a link between the action of this protein and AD [64]. Notably, when *NFKB1* gene is chronically over-expressed, both β-secretase and γ-secretase activities increase, accelerating Aβ production. Conversely, inhibiting NF-κB signaling lowers γ-secretase components and Aβ formation [64]. Notably, increased Aβ levels promote the release of proinflammatory cytokines, which trigger the NF-κB pathway, ultimately providing a positive feedback loop that amplifies Aβ production and sustains neuroinflammation [74]. In this context, physical activity is also highly beneficial. Indeed, the literature data highlights how regular aerobic exercise tends to down-regulate the chronic NF-κB activation [75], with some data specifically pointing to a down-regulation of *NFKB1* and its downstream signaling in the hippocampus. This event likely counters Aβ production and reduces neuroinflammation and associated symptoms (i.e., cognitive dysfunctions) that are commonly associated with neurodegenerative conditions such as AD [75].

Finally, *RYR2* is expressed in neurons and has been shown to be dysregulated in multiple AD models. In this context, it appears that Aβ42 oligomers can increase RYR2-mediated Ca^2+^ release, leading to intracellular Ca^2+^ overload. This dysregulation contributes to circuitry dysfunction and impaired memory acquisition, which are closely associated with pathological mechanisms often observed in neurodegenerative disorders [76].

Notably, the literature data evidenced contradictory observations about *RYR2* expression in AD models. The basis behind these results is likely linked to a variable regulation of *RYR2* along AD pathology development and between brain areas. Indeed, a reduction in RYR2 expression was observed upon treatment with Aβ oligomers [77]. Additionally, *RYR2* expression was shown to be elevated in hippocampal regions in cases with early neurofibrillary pathology and reduced in the subiculum, and CA1-CA4 regions of the late stages [78]. Interestingly, RYR2 seems to be able to interact with presenilins [78], and some studies report that inhibition of RYRs encoded proteins reduces β- and γ-secretase activities [79]. These observations further support its potential correlation with AD. In the context of physical activity, no studies investigated the effect of exercise on brain levels of RYR2. A study on murine models, highlighted an increase in RYR2 in cardiac tissue after exercise [70]. Nevertheless, more data would be needed to evaluate the existence of a link between *RYR2* expression and physical activity.

### 4.2. Effects of Physical Exercise on the Regulation of Amyloid Precursor Protein Expression

While it is largely known that APP mutations are associated with AD, the physiological role of this protein is yet to be completely characterized. The literature data highlight a strong correlation with neuronal functions: the soluble product of α-secretase cleavage has been demonstrated to have neuroprotective, neurotrophic, and synaptogenic properties. Moreover, it is relevant for the long-term potentiation (LTP) process and stimulates neuronal differentiation [80]. There are several other functions related to APP and its cleaved forms: APPα can interact with GABA1B receptors, Na+/K+ ATPases, nAChR, and NMDA receptors (GLUN1 and GLUN2) [81]. Interestingly, recent data have also underlined a role for the monomeric result of APP cleavage by β-secretase. Although the literature evidence is discordant, it seems that APPβ has a physiological role in the brain [27,82,83], especially for glial differentiation [84]. According to the literature data, APP overproduction results in an increase in Aβ concentration (a quantitative increase rather than the qualitative alteration seen in the familial form of AD) [85]. In this context, it should be noted that some of the effects of *APP* overexpression seem to be Aβ independent, probably linked to the increase in the soluble APPs (α and β) and the AICD signal. The expression of *APP* appears to be finely regulated, and several elements take part in this regulation [86,87,88,89].

Physical activity represents a promising intervention to counteract Aβ-induced neurodegeneration by targeting multiple molecular pathways that link both Aβ pathology and exercise-mediated neuroprotection. In this context, while it appears that physical activity mainly influences secretases’ function, it is also able to regulate the expression of the *APP* gene. It has been observed that in a rat model of AD, 4 weeks of treadmill running significantly reduced the Aβ-induced increase in hippocampal *APP*-encoded protein levels in the dentate gyrus and CA1 regions—compared to sedentary rats. This observation suggest that exercise can regulate *APP* expression in a beneficial way, to counter the pathological increase after Aβ exposition [90].

Nevertheless, the specific biological network involved in such effect of physical activity is less clear. Thus, we focused on potential genes whose encoded proteins have been associated with APP regulation. To further increase the specificity of our investigation, we focused only on genes that were dysregulated in AD patients, based on the data contained in the Expression Atlas from EMBL-EBI database. According to our selection (see methods section), we selected genes that were significantly dysregulated in the entorhinal cortices of AD patients compared to controls. Then, we investigated whether such elements can be regulated by physical activity. Table 4 reports the genes down- and up-regulated associated with APP metabolism and expression regulation, and Table 5 reports their correlation with AD and physical exercise according to the literature. To the best of our knowledge, the other genes listed in Table 5 have not been previously studied in relation to physical activity. Future research may help clarify their potential involvement in response to exercise.

Oxidative stress regulation also connects Aβ toxicity and physical activity through key antioxidant enzymes.

Catalase (CAT), a hydrogen peroxide detoxifying enzyme, is increased in the brain by both maternal and adult physical exercise, preventing cognitive deficits induced by early-life stress or colchicine-induced Aβ accumulation [99,100]. While it does not directly regulate *APP* gene expression, CAT effects on ROS regulation may in turn modulate ROS-induced factors (such as NF-κB) and thus exert some control on *APP* expression, since it is known that such factors can up-regulate *APP* [88].

The JAK1 pathway also integrates the effects of Aβ and exercise: Aβ activates the IL-6/JAK1/STAT3 pathway, promoting gliosis and neuroinflammation, whereas physical activity stimulates the IL-4/JAK1/STAT6 axis, driving anti-inflammatory microglial polarization and improving neurological outcomes in ischemic models [92,93].

Finally, *PPP2CA* encodes the catalytic subunit of protein phosphatase 2A (PP2A), a serine/threonine phosphatase. It has been observed that PP2A is able to regulate APP processing, likely through a combination of multiple effects and functions. For example, PP2A dephosphorylates APP at Thr-668, a modification that is important for this protein cleavage [94]. Reduced PP2A methylation and/or activity promotes the accumulation of both phosphorylated tau and APP isoforms and increased secretion of β-secretase-cleaved APP fragments and Aβ peptides. This leads to the accumulation of dephosphorylated tau and APP species and increased secretion of neuroprotective α-secretase-cleaved APP fragments [94]. Additionally, the inhibition of PP2A promotes the axonal accumulation of β-CTF APP fragments by inducing microtubule destabilization and deficits in APP transport [94]. In the context of physical activity, studies in murine models evidenced how exercise is able to increase PP2A, proposing another molecular explanation of the beneficial effects of exercise in AD [95].

### 4.3. Effects of Physical Exercise on Amyloid Clearance

The literature employs several distinct biochemical descriptors for Aβ, which have different pathological and mechanistic implications: (i) monomeric Aβ peptides (the immediate products of APP cleavage), (ii) soluble oligomeric assemblies (small, diffusible aggregates often considered the most synaptotoxic species, with Aβ1–42 oligomers frequently implicated in neuronal dysfunction), (iii) protofibrils (intermediate aggregates), and (iv) insoluble fibrils that constitute extracellular plaques detected histologically or by amyloid PET.

So far, we have discussed genes and pathways related to secretases and APP regulation. Nevertheless, other processes may also be implicated with pathological Aβ accumulation. Several data suggest the prion like behavior of soluble APPβ [101,102]. Physiologically, this behavior may be targeted on creating deposits of monomers for a later use and avoid monomers accumulation [101,102]. This process is finely regulated and balanced by disaggregating, catabolic, and clearance-related processes to prevent abnormal Aβ aggregation increase. Impairments of such processes could lead to pathological Aβ accumulation. Although there are currently no definite proof of disaggregation happening in the brain, it has been found that some enzymes are physiologically able to disaggregate Aβ polymers. HTRA1, which works as an omotrimer, has such a property. The gene *HTRA1* (HtrA Serine Peptidase 1) encodes for a member of the trypsin family of serine proteases. This protein seems to be involved in the regulation of insulin-like growth factors (IGFs) availability by cleaving IGF-binding proteins. It has also been suggested to be a regulator of cell growth. The literature data reports its capacity in dissolving Aβ complexes [103,104] and it has been observed that HTRA1 is able to degrade various fragments from APP cleavage [105]. According to these observations, down-regulation of *HTRA1* expression may impair the encoded protein physiological function, leading to an accumulation of Aβ. Thus, the investigation of its regulatory network may provide new insights into AD etiopathology. Regarding Aβ clearance, the processes mainly involved include ubiquitination, autophagy, phagocytosis, and transport from the brain to the blood via the BBB, arachnoid villi and blood-CSF barrier [106].

Physical activity may exert its beneficial effects on AD by also influencing mechanics related to Aβ clearance and plaques disaggregation. While there is currently no direct evidence that physical activity modulates *HTRA1* expression, it has been observed that physical activity influences clearance. In experimental models of AD, treadmill exercise enhances both central and peripheral Aβ clearance mechanisms, including increased expression of neprilysin and low-density lipoprotein receptor-related protein-1 (LRP1), which are involved in Aβ degradation and transport [13]. Additionally, a 24-week resistance exercise intervention in older adults showed promising effects in reducing brain Aβ accumulation [46]. These findings highlight that physical exercise promotes Aβ clearance through multiple biological pathways, suggesting its potential for AD prevention and management.

Nevertheless, the biological networks bridging physical activity and clearance mechanisms are yet to be completely characterized. In this section, we focused on potential genes whose encoded proteins have been associated with clearance and/or Aβ catabolism. As in the previous sections, we focused only on genes that were dysregulated in AD patients, based on the data contained in the Expression Atlas from EMBL-EBI database. Moreover, we also investigated Heat Shock Proteins (HSP) that, while they were not dysregulated in the public database we used, the literature data highlighted their influence on Aβ clearance and AD. Table 6 reports the genes down- and up-regulated associated with Aβ clearance, and Table 7 reports their correlation with AD and physical exercise according to the literature. Currently, there is no evidence linking the other genes reported in Table 7 to physical activity. Further research may provide insight into their possible role in exercise-induced adaptations.

Overall, the main intersection between Aβ clearance and degradation were represented by apolipoproteins. In particular, Apolipoprotein CIII (APOC3), a small exchangeable apolipoprotein primarily associated with very low-density lipoproteins, has emerged as a key peripheral binder of Aβ peptides. Proteomic profiling of plasma from individuals with mild cognitive impairment and family history of AD revealed that APOC3 copurifies with Aβ oligomers, and low circulating APOC3 levels correlate with both increased Aβ burden and higher AD risk [109,110]. In parallel, two experimental studies have demonstrated that APOC3 expression is responsive to physical activity: long-term endurance training in middle-aged adults led to a significant reduction in APOC3 mRNA in skeletal muscle [111], while a separate trial of combined aerobic and resistance exercise in older individuals reported decreased plasma APOC3 concentrations postintervention [134], suggesting that modulation of APOC3 by exercise may influence peripheral Aβ clearance dynamics.

Complement also seems to be linked to both exercise and Aβ clearance. The classical complement components C1q A chain (C1QA), C1q B chain (C1QB), and the associated serine protease C1s play pivotal roles in tagging fibrillar Aβ for microglial uptake: C1q binds directly to Aβ plaques, enhancing opsonization and subsequent phagocytosis, while C1smediated cleavage of C4 yields C4b fragments that further “decorate” Aβ deposits and promote clearance [117,121]. Interestingly, C1QA enhances microglial activation, amplifying Aβ-induced secretion of proinflammatory cytokines and exacerbating neuroinflammation in affected brain areas [135]. In contrast, C1QB shows a different pattern, as it is down-regulated in a comparative CSF proteome study in AD patients [136]. Intriguingly, physical activity appears to modulate this complement-mediated clearance axis: in both young and aged rodents, voluntary wheel running and treadmill exercise reduce the number of C1qA- and C1qB- positive microglia in cortex and hippocampus [118,120], while in humans, acute endurance and resistance bouts transiently elevate plasma C1s levels [119] but habitual training is associated with lower baseline complement activation, including reduced C1q and C1s concentrations [122], suggesting that exercise both acutely engages and chronically fine-tunes complement dynamics to support Aβ homeostasis.

Finally, a family of proteins crucial for cellular protein quality control, the molecular chaperones known as Heat Shock Proteins (HSPs), are also involved in Aβ degradation and are regulated by exercise. HSPs, particularly Chaperons HSP60, HSP70, and HSP90, are central components of the cellular proteostasis network. Chaperon HSP70 [137,138,139], and to some extent chaperon HSP60 [139] and chaperon HSP90 [140], are of notable relevance in AD and have been increasingly implicated in the clearance of amyloidogenic proteins. These chaperones not only prevent Aβ aggregation but also promote its degradation by facilitating proper folding and directing misfolded peptides toward proteasomal or lysosomal pathways. For instance, chaperon HSP60 has been shown to bind intracellular Aβ and assist in its mitochondrial degradation [124], while chaperon HSP70 enhances microglial phagocytosis of Aβ and mitigates its neurotoxic effects [126]. Chaperon HSP90, in turn, modulates the stability and activity of several Aβrelated signaling proteins, and its inhibition has been associated with enhanced Aβ clearance via increased autophagic flux [127]. Additionally, several lines of evidence suggest that an altered chaperon HSP70 function contributes to neurodegeneration [141,142]. In AD transgenic mouse models, increased chaperon HSP70 expression has been associated with neuroprotective effects [141,142], conferring resistance to apoptosis-inducing stimuli—an effect that is compromised when chaperon HSP70 is down-regulated [143,144].

Notably, physical exercise is a potent modulator of HSPs expression: both acute and chronic aerobic activity significantly up-regulate chaperon HSP70 and chaperon HSP90 in brain and muscle tissues, enhancing cellular stress resilience and protein quality control [125], while resistance training has been reported to elevate circulating chaperon HSP60 levels in older adults, reflecting a systemic stress-adaptive response [128]. Together, these findings suggest that the exercise-induced boost in heat shock protein activity may contribute to improved Aβ clearance and neuronal protection in AD.

**Figure 2:**
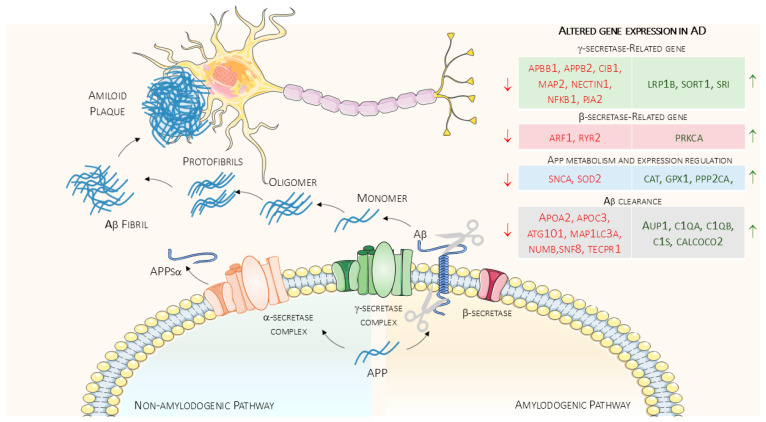
Key Steps of Amyloid Metabolism and Exercise-Responsive Genes. In this figure are reported as the key steps of Aβ metabolism and the genes whose expression is altered in AD patients, and that may pose as link between amyloid pathway and physical exercise, as discussed in the text. Arrows on the right-hand side of the figure indicate whether the genes were down- or up- regulated in the entorhinal cortices of AD patients. The image was created using the image bank of Servier Medical Art (available online: http://smart.servier.com/ accessed on 24 September 2025), licensed under a Creative Commons Attribution 4.0 International License (available online: https://creativecommons.org/licenses/by/4.0/ accessed on 24 September 2025).

In summary, several genes may act as a bridge between physical exercise with Aβ metabolism at different levels. Figure 2. Summarizes the main correlations discussed.

**Figure 3:**
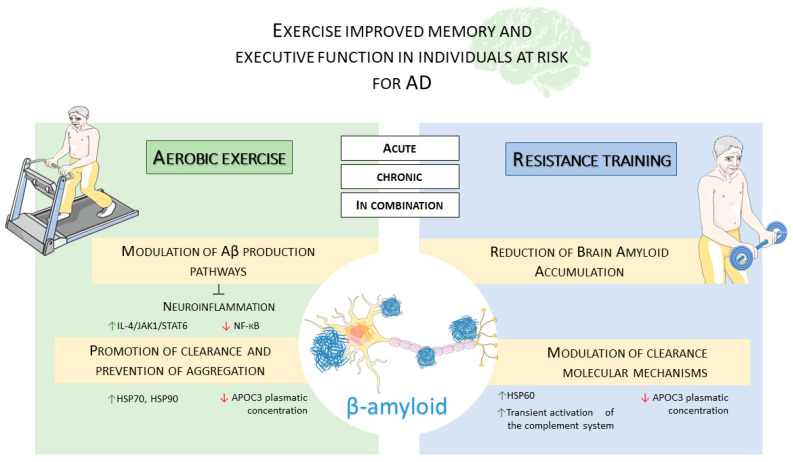
Effect of different types of exercise on Amyloid pathways. This figure illustrates the distinct and overlapping mechanisms through which aerobic exercise and resistance training influence Aβ metabolism and clearance. Aerobic exercise (**left panel**) modulates Aβ production pathways and reduces neuroinflammation by increasing the IL-4/JAK1/STAT6 pathway and decreasing the NF-κB pathway. It also promotes clearance by increasing levels of molecular chaperones like HSP70 and HSP90. Resistance training (**right panel**) reduces brain amyloid accumulation and modulates clearance mechanisms by increasing the molecular chaperone HSP60. Both forms of exercise decrease the plasmatic concentration of APOC3 and lead to an increase in molecular chaperones, as well as a transient activation of the complement system. ↑: Upregulation/Increase; ↓: Downregulation/Decrease; ⟂: Blocking/Inhibition. The image was created using the image bank of Servier Medical Art (available online: http://smart.servier.com/ accessed on 25 September 2025), licensed under a Creative Commons Attribution 4.0 International License (available online: https://creativecommons.org/licenses/by/4.0/ accessed on 24 September 2025).

Finally, in Figure 3, we report on how physical exercise influences Aβ production.

## 5. Conclusions and Future Perspectives

In this review, we summarized the main genetic and molecular pathways influenced by physical exercise that are involved in the regulation of Aβ production, aggregation, and clearance. Genes such as *APP*, *PSEN1*, *BACE1*, *APOE*, and others—including various components of the autophagy and antioxidant systems—were identified as key modulators of Aβ metabolism and neuroinflammation. Many of these genes are differentially expressed or functionally altered in AD and are likewise responsive to physical activity. These findings support the hypothesis that exercise exerts neuroprotective effects, at least in part, by modulating gene expression and molecular pathways converging on Aβ homeostasis.

While direct causality has yet to be established, our integrative approach, which connects data from the literature, gene expression atlases, and protein interaction networks, provides a compelling rationale for considering physical activity as a potential modulator of molecular targets relevant to AD.

Future studies are necessary to validate these associations and mechanistic links through longitudinal and interventional designs. Research integrating genomic, transcriptomic, and proteomic data with physical performance parameters and cognitive outcomes will be critical. Furthermore, the heterogeneity observed in human trials—likely due to differences in exercise modalities, intensities, and individual genetic backgrounds—must be addressed through precision-medicine approaches.

In conclusion, we propose that a targeted evaluation of exercise-responsive genes and their role in Aβ dynamics may pave the way for novel preventive strategies and complementary therapies against AD. Rigorous experimental validation will be required to translate these molecular insights, especially for genes not yet investigated in the context of physical activity, into clinically actionable interventions.

## Figures and Tables

**Table 1:** AD epidemiological data.

Parameters	Time	Metrics		
		Mean	Upper	Lower
DALYs (Disability-Adjusted Life Years)	2021 (2025)	36′332′686.74 (40′363′865.03)	76′873′276.22 (85′077′305.86)	17′237′624.04 (19′031′052.83)
YLDs (Years Lived with Disability)	2021 (2025)	11′582′108.01 (13′150′993.85)	15′296′793.45 (17′401′164.78)	7′961′941.52 (8′999′781.94)
Incidence	2021 (2025)	56′856′688.21 (64′409′200.27)	64′977′511.92 (73′637′607.71)	49′382′064.01 (56′004′781.73)
Prevalence	2021 (2025)	56′856′688.21 (64′409′200.27)	64′977′511.92 (73′637′607.71)	49′382′064.01 (56′004′781.73)

Data for year 2021 collected from [4]. Projections for year 2025 are reported within brackets.

**Table 2:** Genes with altered expression in AD patients’ entorhinal cortices (Secretases).

α-secretase	
Down-regulated genes:	*ANAPC10*, *COCH*, *EGFR*, *HKDC1*, *NHEJ1*, *NUP98*, *PIK3CA*, *SNRPD2*, *TBC1D8*
Up-regulated genes:	*LTA4H*, *PRPF3*
β-secretase	
Down-regulated genes:	*ARF1*, *CIT*, *CRELD1*, *DIAPH1*, *ITIH3*, *MRPL53*, *PRKCB*, *PRRT3*, *RAC3*, *RPGR*, *RYR2*
Up-regulated genes:	*ARHGDIB*, *ECHDC3*, *FLNC*, *PRKCA*, *RHOC*, *SH3BGRL3*, *STX2*, *TECR*
γ-secretase	
Down-regulated genes:	*APBB1*, *APBB2*, *BRSK2*, *CIB1*, *COG3*, *CRHBP*, *DAP3*, *DNER*, *EIF4A3*, *FAF1*, *FBXW9*, *MAP2*, *MLLT11*, *MT-ATP8*, *NECTIN1*, *NFKB1*, *PDXP*, *PJA2*, *RALBP1*, *SERPINI1*, *SF3B5*, *SYAP1*, *TNPO3*, *U2AF2*
Up-regulated genes:	*CHMP4B*, *LRP1B*, *MVP*, *RIPK2*, *SORT1*, *SOX10*, *SRI*, *SUN2*

In the table, we report all the dysregulated genes involved in the regulation of secretases’ activity. The expression data was obtained from the Expression Atlas resource of the EMBL-EBI database.

**Table 3:** Molecular crosstalk between secretase—related genes and physical activity.

Gene	Role in the Context of Aβ	Role in the Context of Physical Exercise
*APBB1*	Adaptor for APP intracellular domain as transcriptional activator; FE65–APP signaling affects memory [52,53].	Not reported
*APBB2*	Regulates synaptic development via APP, the precursor of Aβ [54].	Down-regulated by high and moderate physical activity [55].
*ARF1*	Regulates BACE1 transport and Aβ production [56].	Up-regulated by endurance exercise [57].
*CIB1*	Inhibits γ-secretase, lowering Aβ production in neurons from early AD patients [58].	Not reported
*LRP1B*	Reduces Aβ production [56] and is a substrate of γ-secretase [59].	Not reported
*MAP2*	Aβ1-42 reduces *MAP2* expression in central nervous system (CNS) [60,61].	Physical activity contributes to *MAP2* expression up-regulation [62].
*NECTIN1*	PS1-dependent cleavage links adhesion to Aβ control [63].	Not reported
*NFKB1*	Increases both β- and γ-secretase activity, accelerating Aβ production [64].	Transiently elevated in adipose after exercise [65].
*PJA2*	Lowers APP mRNA via P2X receptor regulation [66].	Not reported
*PRKCA*	PKCα activity mediates Aβ-induced synaptic depression [67].	Not reported
*RYR2*	Ca^2+^ release via *RYR2* enhances β-secretase activity [68], and Aβ promotes RYR2 Ca^2+^ release [69].	*RYR2* expression is up-regulated by exercise [70].
*SORT1*	Regulates APP/Aβ trafficking, accumulates in plaques [71].	Not reported
*SRI*	It counters Aβ and Tau related toxicity [72].	Not reported

In Table 3, the literature data on the dysregulated genes potentially linked with Secretases regulation are summarized. Here, we report the findings supporting the genes activity in the context of secretases regulation and the observations that support a regulation of their expression and/or activity by physical exercise. *APPB1*, *APPB2*, *ARF1*, *CIB1*, *MAP2*, *NECTIN1*, *NFKB1*, *PJA2*, and *RYR2* are down-regulated in AD, while *LRP1B*, *PRKCA*, *SORT1*, *SRI* are up-regulated. Please note that only genes with literature support associating them with secretases are reported in the table.

**Table 4:** Genes with altered expression in AD patients’ entorhinal cortices (APP regulation).

*APP Metabolism and Expression Regulation*	
Down-regulated genes:	*APBB1*, *PSAP*, *RFC2*, *RFC4*, *RFC5*, *SNCA*, *SOD2*, *TOR1A*, *WDR12*
Up-regulated genes:	*CAT*, *GPX1*, *JAK1*, *LAMP2*, *PPP2CA*, *WBP11*

In the table, we report all the dysregulated genes involved in the regulation of APP metabolism. The expression data was obtained from the Expression Atlas resource of the EMBL-EBI database.

**Table 5:** Molecular crosstalk between APP-related regulating genes and physical activity.

Gene	Role in the Context of Aβ	Role in the Context of Physical Exercise
*CAT*	May indirectly regulate APP expression by modulation of ROS, and the subsequent regulation of the ROS-induced activation of NF-κB/AP-1 [88].	Physical activity elevates CAT expression in murine models [91].
*GPX1*	GPX1 also indirectly regulates APP expression by acting on ROS concentration [88].	Not reported
*JAK1*	Activated by Aβ via IL-6/JAK1/STAT3, promoting gliosis and neuroinflammation [92].	Exercise stimulates IL-4/JAK1/STAT6, driving anti-inflammatory microglial polarization and neuroprotection [93].
*PPP2CA*	Component of PP2A. It is associated with decreased concentration of Aβ peptides, due to its modifications on APP [94].	Exercise in murine AD models significantly increases PP2A, likely exerting positive effects in such models [95].
*SNCA*	Participates in synaptic dysfunction and Lewy body formation in presence of Aβ and tau aggregates [96].	Not reported
*SOD2*	Down-regulated in Aβ-exposed neural stem cells, increases oxidative damage. Its increase seemingly mitigates Aβ plaque burden in Murine models [97,98].	Not reported

In Table 5, the literature data on the dysregulated genes potentially linked with APP metabolism and expression regulation are summarized. Here, we report the findings supporting the genes activity in the context of APP metabolism and the observations that support a regulation of their expression and/or activity by physical exercise. *SNCA* and *SOD2* are down-regulated in AD, while *CAT*, *GPX1*, *JAK1*, and *PPP2C* are up-regulated. Please note that the only genes with literature support in the context of APP metabolism and/or expression regulation are reported in the table.

**Table 6:** Genes with altered expression in AD patients’ entorhinal cortices (Aβ Clearance).

*Aβ Clearance*	
Down-regulated genes:	*APOA2*, *APOC3*, *ATG101*, *BSN*, *COMMD9*, *COX4I1*, *CTSF*, *GABARAP*, *MADD*, *MAP1LC3A*, *NUMB*, *NUMBL*, *SNF8*, *TAOK2*, *TECPR1*, *UBE2H*, *VPS36*, *ZFYVE20*
Up-regulated genes:	*ANKFY1*, *ANP32A*, *APPL2*, *AUP1*, *C1QA*, *C1QB*, *C1S*, *CALCOCO2*, *CD99*, *CNPY4*, *FHL5*, *MAP2K6*, *MAVS*, *TAOK3*

In the table we report all the dysregulated genes involved in the regulation of Aβ clearance. The expression data was obtained from the Expression Atlas resource of the EMBL-EBI database.

**Table 7:** Molecular crosstalk between Aβ clearance-related genes and physical activity.

Gene	Role in the Context of Aβ	Role in the Context of Physical Exercise
*APOA2*	In an experimental model, APOA2(b) suppresses Aβ fibril extension [107]. APOA2(c) promotes systemic and spontaneous Aβ deposition in transgenic mice [108].	Not reported
*APOC3*	APOC3 has been shown to bind Aβ, and low levels of this protein are associated with AD risk [109,110].	In murine experimental models it was observed that treadmill exercise increases APOC3 [111]; however, another study highlighted a reduction in APOC3 after aerobic exercise [112].
*ATG101*	ATG101 is an essential component of the ULK1–ATG13–FIP200 initiation complex, which is important for autophagy [113]. Autophagy has been implicated in proper APP and Aβ turnover [114]. Its down-regulation in AD brains likely contributes to the autophagy defects that exacerbate Aβ accumulation.	Not reported
*AUP1*	While no direct correlation of Aβ degradation has been observed, this protein is an important component of the HRD1-SEL1L complex, essential in the degradation of misfolded proteins of the plasmatic reticulum, where the highly amyloidogenic Aβ1-42, is generated and accumulated [115,116].	Not reported
*C1QA*	Opsonizes Aβ fibrils for microglial uptake, potentially promoting plaque clearance, even though the process is slow [117]. It is also involved in neuroinflammation [117].	Exercise seemingly decreases C1QA+ microglia in murine models [118,119].
*C1QB*	Opsonizes Aβ fibrils for microglial uptake, potentially promoting plaque clearance, even though the process is slow [117]. It is also involved in neuroinflammation [117].	Exercise reduces C1q+ microglia [118,120], even though some data indicate that acute exercise increases C1QB [120].
*C1S*	Participates in the opsonization of Aβ fibrils for microglial uptake, potentially promoting plaque clearance, even though the process is slow [117,121].	Up-regulated in response to ultra-endurance running and resistance training [122].
*CALCOCO2*	Up-regulated in AD mouse brain; binds intracellular Aβ in autophagic vesicles for lysosomal degradation [123].	Not reported
*Chaperon HSP60*	Inhibits Aβ1–40 and Aβ1–42 aggregation, reducing toxic oligomer formation and supporting mitochondrial function [124].	In murine experimental models of AD exercise increased the expression of Chaperon HSP60 [125].
*Chaperon HSP70*	Prevents Aβ aggregation by promoting solubilization and degradation via autophagy and proteasomal pathways, protecting neurons from Aβ-induced toxicity [126].	In murine experimental models of AD exercise increased the expression of Chaperon HSP70 [125].
*Chaperon HSP90*	Binds to Aβ fibrils and tau aggregates, modulating assembly/disassembly and contributing to Aβ pathology regulation [127].	Appears to be up-regulated by exercise, despite it having variable responses to specific exercise types and modalities [128].
*MAP1LC3A*	Multiple studies report decreased expression of LC3 proteins—including LC3A—in postmortem AD hippocampus and cortex, correlating with autophagic-vacuole accumulation and extracellular Aβ plaque buildup [129].	Not reported
*NUMB*	Likely involved in the transport of APP and in the modulation of its accumulation [130].	Not reported
*SNF8*	SNF8 encodes the EAP30 subunit of the ESCRTII complex. Loss of its function impairs lysosome trafficking, promoting aberrant accumulation of Aβ and damage, as observe in yeast models of AD [131,132].	Not reported
*TECPR1*	Results down-regulated in murine models of AD. Here, the induced overexpression of TECPR1 seems able to restore autophagic flux, reducing abnormal proteins accumulation [133].	Not reported

In Table 7 are summarized the literature data on the dysregulated genes potentially linked with Aβ Clearance. Here we report the findings supporting the genes’ activity in the context of clearance and the observations that support a regulation of their expression and/or activity by physical exercise. *APOA2*, *APOC3*, *ATG101*, *MAP1LC3A*, *NUMB*, *SNF8*, and *TECPR1* are down-regulated in AD, while *AUP1*, *C1QA*, *C1QB*, *C1S*, and *CALCOCO2* are up-regulated. Please note that only gene with literature support in the context of Aβ Clearance are reported in the table.

## Supplementary Materials

The following supporting information can be downloaded at: https://www.mdpi.com/article/10.3390/ijms26199816/s1.

## Abbreviations

The following abbreviations are used in this manuscript:

ABCA7	ATP binding cassette subfamily A member 7
AD	Alzheimer’s Disease
ANAPC10	Anaphase Promoting Complex Subunit 10
ANKFY1	Ankyrin Repeat And FYVE Domain Containing 1
ANP32A	Acidic Nuclear Phosphoprotein 32 Family Member A
APBB1	Amyloid Beta Precursor Protein Binding Family B Member 1
APBB2	Amyloid Beta Precursor Protein Binding Family B Member 2
APOA2	Apolipoprotein A-II
APOC3	Apolipoprotein CIII
APOE	Apolipoprotein E
APP	Amyloid Precursor Protein
APPL2	Adaptor Protein, Phosphotyrosine Interacting With PH Domain And Leucine Zipper 2
ARF1	ADP-Ribosylation Factor 1
ARHGDIB	Rho GDP Dissociation Inhibitor Beta
ATG101	Autophagy Related 101
AUP1	AUP1 Lipid Droplet Regulating VLDL Assembly Factor
Aβ	Amyloid-β
BACE1	Beta-Site Amyloid Precursor Protein Cleaving Enzyme 1
BBB	Blood–Brain Barrier
BIN1	Bridging Integrator 1
BRSK2	BR Serine/Threonine Kinase 2
BSN	Bassoon Presynaptic Cytomatrix Protein
C1QA	Complement C1q A Chain
C1QB	Complement C1q B Chain
C1S	Complement C1s
CALCOCO2	Calcium Binding And Coiled-Coil Domain 2
CAT	Catalase
CD99	CD99 Molecule
CHMP4B	Charged Multivesicular Body Protein 4B
CIB1	Calcium And Integrin Binding 1
CIT	Citron Rho-Interacting Serine/Threonine Kinase
CLU	Clusterin
CNPY4	Canopy FGF Signaling Regulator 4
CNS	Central Nervous System
COCH	Cochlin
COG3	Component Of Oligomeric Golgi Complex 3
COMMD9	COMM Domain Containing 9
COX4I1	Cytochrome C Oxidase Subunit 4I1
CR1	Complement Receptor 1
CRELD1	Cysteine Rich With EGF Like Domains 1
CRHBP	Corticotropin Releasing Hormone Binding Protein
CTSF	Cathepsin F
DALYs	Disability-Adjusted Life Years
DAP3	Death Associated Protein 3
DIAPH1	Diaphanous Related Formin 1
DNER	Delta/Notch Like EGF Repeat Containing
ECHDC3	Enoyl-CoA Hydratase Domain Containing 3
EGFR	Epidermal Growth Factor Receptor
EIF4A3	Eukaryotic Translation Initiation Factor 4A3
EOAD	Early-Onset Alzheimer’s Disease
FAF1	FAS Associated Factor 1
FBXW9	F-Box And WD Repeat Domain Containing 9
FHL5	Four And A Half LIM Domains 5
FLNC	Filamin C
FOXO1/3	Forkhead Box Protein O1/3
GABARAP	GABA Type A Receptor-Associated Protein
GPX1	Glutathione Peroxidase 1
GWAS	Genome-Wide Association Studies
HKDC1	Hexokinase Domain Containing 1
HSP	Heat Shock Protein (including HSP60, HSP70, HSP90)
HTRA1	HtrA Serine Peptidase 1
IL-4/IL-6	Interleukin 4/6
ITIH3	Inter-Alpha-Trypsin Inhibitor Heavy Chain 3
JAK1	Janus Kinase 1
LAMP2	Lysosomal Associated Membrane Protein 2
LOAD	Late-Onset Alzheimer’s Disease
LRP1	Low-Density Lipoprotein Receptor-Related Protein-1
LRP1B	LDL Receptor Related Protein 1B
LTA4H	Leukotriene A4 Hydrolase
LTP	Long-Term Potentiation
MADD	MAP-kinase activating death domain
MAP1LC3A	Microtubule Associated Protein 1 Light Chain 3 Alpha
MAP2	Microtubule Associated Protein 2
MAP2K6	Mitogen-Activated Protein Kinase Kinase 6
MAVS	Mitochondrial Antiviral Signaling Protein
MLLT11	MLLT11, PHD Finger Containing
MRPL53	Mitochondrial Ribosomal Protein L53
MT-ATP8	Mitochondrially Encoded ATP Synthase Membrane Subunit 8
MVP	Major Vault Protein
NECTIN1	Nectin Cell Adhesion Molecule 1
NFKB1	Nuclear Factor Kappa B Subunit 1
NHEJ1	Non-Homologous End Joining Factor 1
NUMB	Numb Endocytic Adapter Protein
NUMBL	Numb Like Endocytic Adaptor Protein
NUP98	Nucleoporin 98
PRKN	Parkin RBR E3 Ubiquitin Protein Ligase
PD	Parkinson’s Disease
PDXP	Pyridoxal Phosphatase
PIK3CA	Phosphatidylinositol-4,5-Bisphosphate 3-Kinase Catalytic Subunit Alpha
PINK1	PTEN Induced Kinase 1
PJA2	Praja Ring Finger Ubiquitin Ligase 2
PP2A	Protein Phosphatase 2A
PPP2CA	Protein Phosphatase 2 Catalytic Subunit Alpha
PRISMA	Preferred Reporting Items for Systematic Reviews and Meta-Analyses
PRKCA	Protein Kinase C Alpha
PRKCB	Protein Kinase C Beta
PRPF3	Pre-mRNA Processing Factor 3
PRRT3	Proline Rich Transmembrane Protein 3
PSAP	Proactivator Polypeptide
PSEN1	Presenilin 1
PSEN2	Presenilin 2
RAC3	Rac Family Small GTPase 3
RALBP1	RalA Binding Protein 1
RFC2/4/5	Replication Factor C Subunit 2, 4, 5
RHOC	Ras Homolog Family Member C
RIPK2	Receptor Interacting Serine/Threonine Kinase 2
RPGR	Retinitis Pigmentosa GTPase Regulator
RYR2	Ryanodine Receptor 2
SERPINI1	Serpin Family I Member 1
SF3B5	Splicing Factor 3b Subunit 5
SH3BGRL3	SH3 Domain Binding Glutamate-Rich Protein Like 3
SIRT1	Sirtuin 1
SNCA	Synuclein Alpha
SNF8	SNF8, ESCRT-II Complex Subunit
SNRPD2	Small Nuclear Ribonucleoprotein D2 Polypeptide
SOD2	Superoxide Dismutase 2
SORT1	Sortilin 1
SOX10	SRY-Box Transcription Factor 10
SRI	Sorcin
STX2	Syntaxin 2
SUN2	Sad1 And UNC84 Domain Containing 2
SYAP1	Synapse Associated Protein 1
TAOK2/3	TAO Kinase 2, 3
TBC1D8	TBC1 Domain Family Member 8
TECPR1	Tectonin Beta-Propeller Repeat Containing 1
TECR	Trans-2,3-Enoyl-CoA Reductase
TNPO3	Transportin 3
TOR1A	Torsin Family 1 Member A
TREM2	Triggering Receptor Expressed On Myeloid Cells 2
U2AF2	U2 Small Nuclear RNA Auxiliary Factor 2
UBE2H	Ubiquitin Conjugating Enzyme E2 H
VPS36	VPS36, ESCRT-II Complex Subunit
WBP11	WW Domain Binding Protein 11
WDR12	WD Repeat Domain 12
WHO	World Health Organization
YLDs	Years Lived with Disability
YLLs	Years of Life Lost.
ZFYVE20	Zinc Finger FYVE-Type Containing 20
