# Supplementary figures and images for: Gene-Exercise Interactions in Amyloid Metabolism and Clearance: Implications for Alzheimer’s Disease
Source: Int J Mol Sci. 2025 Oct 9;26(19):9816. doi: 10.3390/ijms26199816 (PMC12525307; doi:10.3390/ijms26199816)

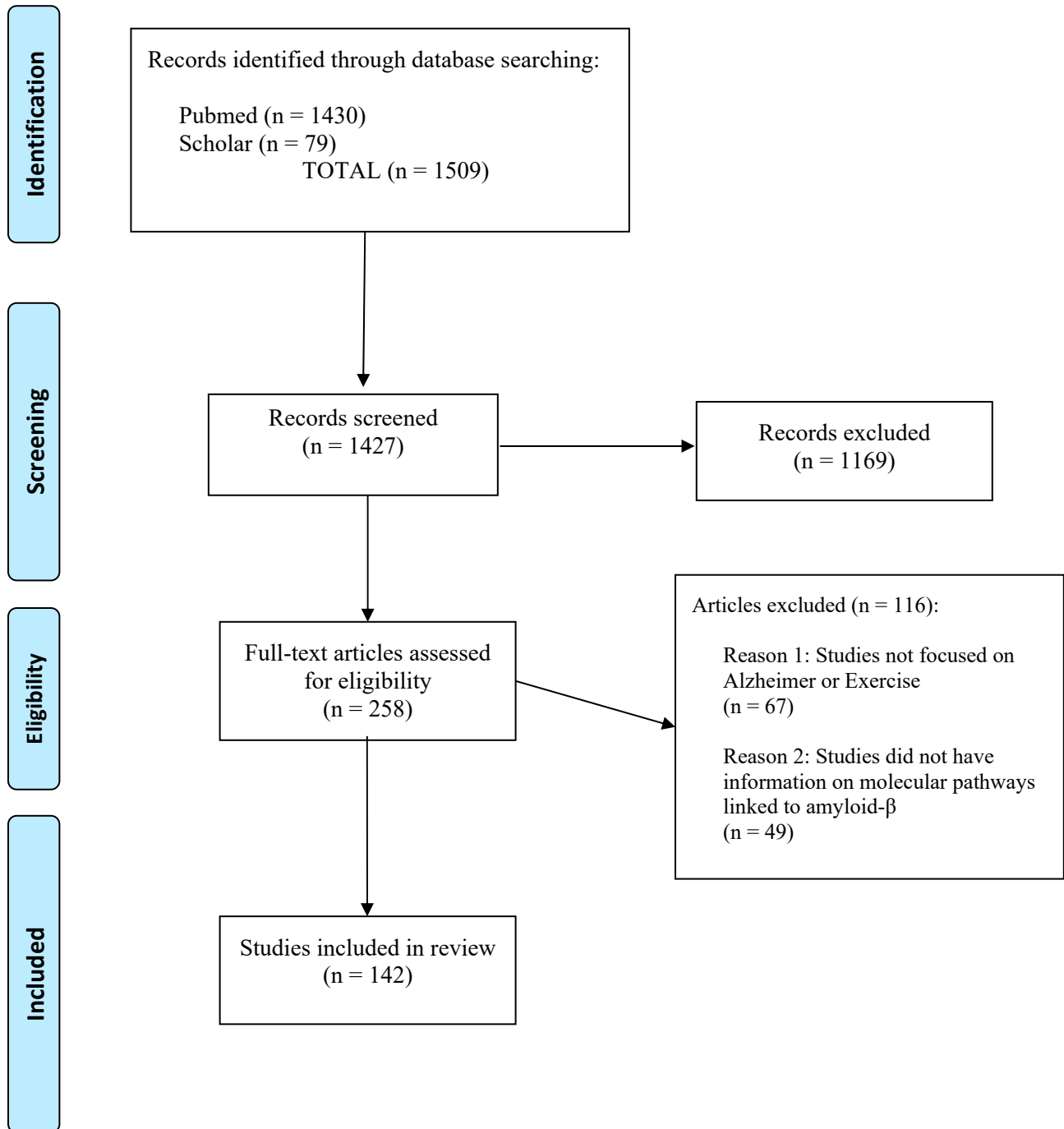

Supplement: Supplementary file 1 [file ijms-26-09816-s001.zip › Supplementary Figure S1.pdf]
